# Integrating Maternal Depression Screening Into an Early Intervention Program: An Implementation Evaluation

**DOI:** 10.1177/10783903221116648

**Published:** 2022-08-09

**Authors:** Rebecca E. Salomon, Julee B. Waldrop, Maureen Baker, Marcia A. Mandel, Doré R. LaForett, Linda S. Beeber

**Affiliations:** 1 Rebecca E. Salomon, PhD, RN, PMHNP-BC, Independent Researcher, Hillsborough, NC, USA; 2 Julee B. Waldrop, DNP, PNP-BC, FNP-BC, CNE, FAANP, FAAN, Duke University, Durham, NC, USA; 3 Maureen Baker, PhD, RN, The University of North Carolina at Chapel Hill, Chapel Hill, NC, USA; 4 Marcia A. Mandel, PhD, Independent researcher, Chapel Hill, NC, USA; 5 Doré R. LaForett, PhD, The University of North Carolina at Chapel Hill, Chapel Hill, NC, USA; 6 Linda S. Beeber, PhD, PMHCNS-BC, FAAN, The University of North Carolina at Chapel Hill, Chapel Hill, NC, USA

**Keywords:** developmental disabilities, family support, health services, women’s mental health

## Abstract

**Background:**

In all 50 states, early intervention (EI) services to improve long-term child cognitive and academic outcomes are provided to infants and toddlers with suspected or diagnosed developmental delays. When mothers of EI-enrolled children experience depressive symptoms, uptake of EI services can be compromised.

**Aims:**

The purpose of the article is to present a depressive symptom screening intervention for mothers consisting of toolkit development for EI staff and families, symptom screening for mothers and follow-up protocol. To formally evaluate the implementation of the intervention, our research team followed the consolidated framework for implementation research (CFIR).

**Methods:**

Participants were 12 EI service coordinators across two offices. Focus groups and individual interviews were used to develop the toolkit and education module. Through the five CFIR domains, we evaluated the implemented intervention in order to allow other teams to learn from our experiences.

**Results:**

Our team successfully partnered with SCs to develop the intended deliverables. Still, the SCs found it challenging to conduct the screenings and reported mixed success.

**Conclusions:**

Preparation of EI SCs to integrate mental health screenings into their existing skillsets requires a high level of support from the research team, resulting in a rich understanding of the barriers—and potential rewards—for staff and families.

Early intervention (EI) services—aimed at improving long-term child cognitive and academic outcomes—are provided to infants and toddlers with suspected or diagnosed developmental delays in all 50 states, with federal grant support available to states since 1975 (*Early Childhood Technical Assistance Center*, n.d.). EI improves long-term infant-toddler adaptation, lowers the cost of care, and is most effective if caregivers actively participate in the provision of services (Barton & Fettig, 2013; North Carolina Department of Health & Human Services, 2002; Spagnola & Fiese, 2007). However, depressive symptoms could reduce caregivers’ ability to provide the daily child development–promoting activities recommended by EI. While there are many types of caregivers who engage in EI services with children, researchers outside of the EI setting have historically focused on the role of the mother and maternal responsivity. In those studies conducted outside of the EI setting, researchers have found that depressive symptoms reduce mothers’ consistent use of developmentally sensitive, child-centered speech (Brennan et al., 2002; Stein et al., 2010) which, in turn, leads to significant language delays and other negative child cognitive and behavioral outcomes (Grace et al., 2003). Given these findings, our team was interested in whether reduced maternal responsivity in the EI setting could increase the child’s risk for communication and behavioral problems, with a future interest in expanding to other caregivers.

Only two studies have addressed the presence of depressive symptoms in mothers of children in EI. One used a large data set from the early childhood longitudinal study’s birth cohort and found that 35% of mothers experienced significant depressive symptoms per self-report measures (Feinberg et al., 2012). The other study found that over a third of mothers of infants and toddlers enrolled in EI had severe depressive symptoms and depression histories when assessed with both self-report and standardized diagnostic interviews (Beeber et al., 2017). In a separate study with a non-EI sample, the same research team also found that when depressed mothers were provided with concrete, attainable skills for improving interactions with their child, the impact of depression on both mother and child was substantially reduced (Beeber et al., 2010, 2013).

Therefore, it could be argued that integration of mental health services with EI could lead to improved maternal mental health and child outcomes. However, the implementation research on the development of such programs is very limited. Alvarez et al. (2015) call for research to inform program development, in particular, to explore the acceptability and feasibility of such programs with EI staff and mothers. One project, *Helping Families Raise Healthy Children*, provided a step-by-step example for developing a toolkit and implementation of screening for mental health symptoms, along with resources for mothers including referrals and integration of mental health services into EI program delivery (Reynolds et al., 2012). Our team followed the Reynolds et al. example and developed a toolkit and protocol tailored to meet the unique needs of this EI program and families.

Service coordinators (SCs) oversee the child’s evaluation and enrollment in EI. They are responsible for developing the individualized family service plan, or IFSP, based on the assessed needs of the child and family. They have regular and ongoing contact with the family, help the family access services, monitor the child’s progress, and help the family transition out of EI (no later than the child’s third birthday). However, they do not systematically address maternal depression or have specialized skills to identify depressive symptoms and engage mothers in seeking help or care for themselves. A previous study conducted by some members of the current research team characterized depressive symptoms in mothers of children in EI as a modifiable factor (McKechnie et al., 2018). During that study, the SCs indicated a need for resources and skills to educate mothers about depression, introduce and complete symptom screens, refer mothers for treatment, and encourage mothers with depressive symptoms to engage in EI service.

Because child-focused services are already being provided on a regular basis in the family’s home, EI is an ideal setting in which to integrate screening, referral, and targeted skills for mothers with depressive symptoms with an intent to improve parent–child interactions and child outcomes. Drawing from Reynolds et al. (2012), our team aimed to address this gap in research and practice by developing a toolkit and protocol for maternal depression screening of mothers whose children are participating in EI. To achieve these goals, we leveraged the parent–SC relationship to implement our newly developed, nonthreatening depression screening toolkit and protocol, along evidence-based referral and resources as needed.

The purpose of this manuscript is to describe a depressive symptom intervention for mothers of children receiving EI services that consists of toolkit development, symptom screening, and follow-up protocol. In addition, this article provides an in-depth description of the implemented intervention using the Consolidated Framework for Implementation Research (CFIR; Damschroder et al., 2009) that will allow other teams to learn from our experiences.

## Methods

### Setting

The two sites for the study were local offices of a single agency delivering EI services in the southeastern United States. The single agency serves eight counties. Within that agency, sites A and site B serve very similar settings with small urban, suburban, and rural areas. Site A served as the site for the development of the education modules and tool kit, including protocol development, and as the initial implementation site. Our team next delivered the education modules and the SCs implemented the screening protocol using the toolkit in site B.

### Participants

Participants were EI SCs at the two offices (sites A and B). At site A, 5 out of the 6 (83%) SCs participated in four, 90-minute focus groups. All had worked at that site for a year or more and one had worked in an EI agency in another state. At site B, 7 (100%) SCs were provided with the education, training, protocol, and toolkit for implementation. Then, after receiving the training, one SC at site B withdrew from the study for personal reasons. Across both sites, all of the SCs presented as women in gender (though we did not ask for a self-report of their gender or sex). The SCs were early middle aged/middle aged. To be hired as SCs in the state where we conducted this study, an individual is currently required to have a bachelor’s degree in a related field as well as professional experience working with the population served. Our team did not collect demographic data due to the small size of the sample and potential for identification of study participants.

### Procedures

Before initiating contact with sites A and B, this project was approved by the institutional review board at a large university in the southeastern United States. Focus groups were conducted with site A SCs to assess their learning needs related to screening mothers for depressive symptoms to inform the development of the educational sessions. At each focus group, the team presented a proposed toolkit to support screening for depression for use by the SCs, which were revised iteratively based on feedback from the participants. Over the course of the intervention, three focus groups lasting approximately 2 hours each were conducted by two to three researchers from the team. Audio recordings from the focus groups were transcribed and analyzed for themes using MaxQDA software. Identified themes were then used to structure the outline and creation of the toolkit, which was shared with the site A SCs during a follow-up to check accuracy and adequacy.

The finalized toolkit included strategies for engaging mothers with symptoms of depression, an algorithm for carrying out the screening, referral and follow-up, documentation and crisis management, and a manualized curriculum for training SCs. Once trained in the use of the toolkit, we expected SCs at site A to be prepared to begin screenings. After development with site A, our team adapted the toolkit with referral resources applicable to site B and trained site B SCs to use the educational module, toolkit, and screening protocol. Data on the number of clients screened, as well as any indicated referrals, were collected from all SCs by the agency Director. SCs’ perceptions of feasibility of screening were discussed in a group and individually with their supervisors, the agency Director, and research team members. SCs also reported the response of the mothers to the materials, screening, and referral resources. Finally, the SCs collected data on the number of screenings for depressive symptoms, maternal depressive symptom scores, the number of referrals made, and the number of referrals accepted by mothers.

### Measures

Beyond theme analysis of the focus group sessions, data were collected on the number of screenings conducted for depressive symptoms, the number of referrals made to psychiatric care, and the number of referrals accepted by mothers using a case summary sheet created for the study. Maternal depressive symptoms were measured through the Patient Health Questionnaire-9 (PHQ-9), a 9-item scale with higher scores indicating higher levels of depressive symptoms with established validity (Kroenke et al., 2001; Martin et al., 2006; Merz et al., 2011; Mitchell et al., 2016). Consistent with questionnaire recommendations, participants who scored higher than 10 on the PHQ-9 were considered to have significant depressive symptoms (Kroenke et al., 2001). In addition, if a participant scored 15 or higher, SCs were prompted to explicitly screen for imminent danger (i.e., participant expressing thoughts or feelings of hurting herself, her child, or someone else) and follow an emergency referral tree described later.

## CFIR Evaluation

To evaluate the implementation of this project, the intervention must be viewed as more than just the actual screening of clients for depressive symptoms. The intervention in this project included the work of developing the education that the SCs needed—and requested—into a modularized presentation and a supporting toolkit which can continue to be used in a sustainable manner with new staff as well as expanded to other agencies in the region, state, and beyond.

**Figure 1.**
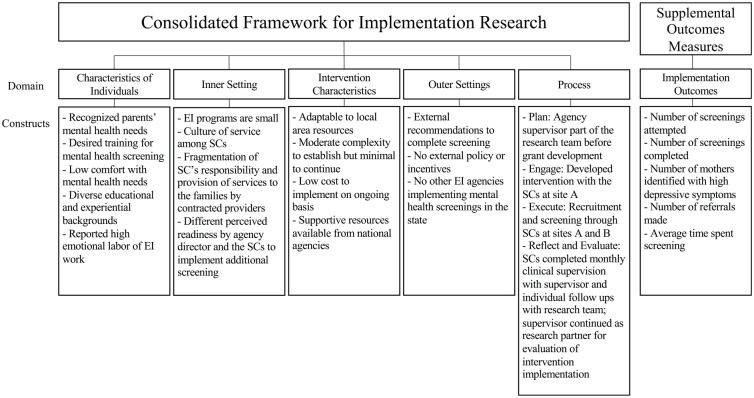
CFIR Evaluation and Implementation Outcomes. *Note*. Reprinted from Tinc et al. (2018), with permission from Elsevier. EI = Early Intervention; SC = service coordinator.

To evaluate our project, we used the CFIR (Damschroder et al., 2009). The purpose of the CFIR is to facilitate formative evaluation of research efforts to put evidence into practice. The framework includes five domains: (a) characteristics of individuals, (b) inner setting, (c) intervention characteristics, (d) outer setting, and (e) process (Damschroder et al., 2009). A representation of our evaluation can be seen in Figure 1.

### Characteristics of Individuals

First, the CFIR framework examines the characteristics of individuals. The SCs at site A believed the proposed intervention was needed to address a recognized problem of mental health needs in parents. They described in rich detail frequently occurring problematic situations with families that were complicated by mental health symptoms. These SCs also identified the need to increase their professional capacity to recognize depressive symptoms in parents. At the same time, however, their evidence-based knowledge, comfort level, and skills regarding identification of mental health issues and their ability to address them was initially low. They expressed concerns about whether they could manage additional responsibilities and whether formal screening and documentation of parents’ symptoms would lead to greater responsibility or personal legal liability. In relation to the intervention, their diverse educational and experiential backgrounds did not provide a consistent baseline level of knowledge, comfort, or skill to support the change in their practice.

In addition, the site A SCs expressed their experiences and concerns about the work they already engaged in with the families. They shared their emotional experiences, including frustration at having limited mental health resources to offer parents and the sorrow they felt when an enrolled child died. They also described the challenges of maintaining their own physical and mental health within the context of their work.

### The Inner Setting

We then followed the CFIR framework and evaluated the inner setting. The federal support for EI services began in 1975 when Public Law 94-142 passed, and this law was eventually replaced by the *Individuals with Disabilities Education Act* (*Early Childhood Technical Assistance Center*, n.d.). However, despite federal support, the scope and intensity of service provided by each EI program is dependent on state and local funding. The number of SCs at each site is small, and there is a service culture with SCs taking responsibility for their cases. However, in this state, the intervention services for children and their families are not provided by the local EI agency but by independent contractors and within the agency are called service providers. Although SCs are responsible for managing the IFSPs and the service providers that delivers the intervention, a few SCs suggested that collaboration with the service providers in an effective manner is challenging due to time and resource constraints as well as the program structure described above. These challenges left some SCs feeling that they had limited power to provide input beyond the follow-up appointments.

At the outset of this project, the agency Director believed there was the capacity of change but the SCs already felt overwhelmed/overworked and their desire for taking on additional responsibilities was low. Their perceived readiness to implement screening was also low. However, the learning climate was high and the opportunity to partner with experts and provide feedback into the process intrigued them, and the agency Director assured them of protected time to engage in the project. This director removed herself from participation in all focus groups and analysis of recordings so the SCs could feel safe to discuss their thoughts and feelings.

The source of the intervention was external to the SCs, though at least in part internal to the EI agency. Implementation of the intervention was supported by the agency director and based on guidelines and recommendations from the American College of Obstetrics and Gynecology and the American Academy of Pediatrics and the National Association of Pediatric Nurse Practitioners.

### Intervention Characteristics

We used the CFIR framework to examine the intervention characteristics. As explained earlier, the research team considers the implemented project to be the entire process of developing the education module and toolkit as well as the delivery of screening by SCs. For future sites interested in implementing a similar screening process, it would be important to factor in both time and financial resources to adapt the existing module and toolkit to the specific agency and catchment area of the EI site.

This initial period of this project (i.e., when the materials were developed and adapted to each site) was the most intensive component of the intervention. In future adaptations, all of the resources produced for this project (i.e., the education module and the toolkits) would need to be updated to identify local area resources. Our experience in working with the SCs suggests that the initial transition into screening is moderately complex and getting SC engagement can be challenging, but that once the screening process is established, there would be minimal complexity to continuing to conduct the intervention. In addition, this intervention was low cost to implement on an ongoing basis, with the biggest expenses being SCs’ time and printing resources.

Within this specific EI agency, the relative advantage over the status quo was not perceived as optimal by the SCs at the beginning of this project. Although initiating a screening tool may sound like a simple intervention, it was perceived to be quite complex by the SCs. For one, they did not feel they had the education or skills to deal with the mothers’ mental health needs. In addition, the SCs perceived their time to be a scarce resource, and they felt the implementation of the screening would take too much time.

### The Outer Setting

The CFIR framework specifies evaluation of the outer setting which, in this instance, was the administrating agencies throughout the state in which EI services were being delivered. Each EI services agency is part of a state program. At the time of this study, no other EI agencies in NC were screening for mental health symptoms in mothers of enrolled children as it is not a required component of service despite being recommended by professional organizations (“ACOG Committee Opinion No. 757: Screening for Perinatal Depression,” 2018; Earls et al., 2019).

### Process

The process domain of the CFIR framework can be broken down into four stages of planning, engaging, executing, and reflecting/evaluating. The EI agency director became a research team member prior to the development of this intervention and was involved in every step of the process, including securing grant funding. Other interprofessional and interdisciplinary team members included psychiatric mental health researchers and nurse practitioners/clinicians, a primary-care pediatric nurse practitioner, and child development experts. At the outset of the project, the research team met with supervisory personnel at site A and determined that the best strategy for moving forward would be engaging the SCs using a focus group approach. This approach was selected so that the SCs were in collaboration with the project team and to improve the SCs comfort and commitment to the screening process.

Once research funding was secured, the intervention planning continued as a partnership between the researchers as the topic experts and the SCs as expert in their roles. In the first and second focus groups, SCs were asked to describe what psychosocial challenges they observed when interacting with parents in all stages of the program. Follow-up questions probed for specific mental health issues like maternal depression. SCs were asked about the barriers encountered when screening for and addressing maternal depression, as well as a wish list of what they needed to help mothers who were facing mental health challenges.

The SCs described the critical nature of EI in the lives of families as they received initial information about their child’s developmental delays or conditions and adjusted to the child’s needs. They described the varying and intense emotional needs of parents of children with developmental delays or conditions, with grief being a process that was universal to parents. SCs described that some parents had pre-existing mental health symptoms, while other parents developed depressive symptoms in the context of grief, increased stressors stemming from the child’s condition, and pre-existing hardships. These were identified as economic insufficiency, lack of social support and lack of access or availability of community resources. In these initial focus groups, the SCs also identified gaps in their knowledge, skills, and attitudes that shaped the development of the educational module.

In the third focus group, the research team shared potential toolkit components such as web-sourced materials and drafts of guidance materials addressing the frequently occurring situations that SCs had identified. These included such topics as grieving, stress management, coping strategies, home safety, and recognizing emergency situations with the child. The SCs were apprehensive about potential mental health crises in parents, especially suicidal ideation. Two of the senior investigators on the research team, along with the agency director, addressed their fears directly through extensive discussion of their role and scope of practice, best practices in suicide and mental health crisis management and the creation of agency protocols for management, communication, and documentation.

Given the concerns of the SCs about screening the participants for depression, particular attention was given to providing support for that task. For example, the toolkit included scripts to guide the SCs in broaching the topic of mental health screening with parents. Our team also worked with the agency director to create two decision aids—one for screening and referral and one for crisis decision—which were developed to fit the unique needs of the SCs at the sites. These included screening thresholds for referral and consultation with agency supervisory staff, referral protocols, and documentation protocols. In the case of a positive screen, the SCs were well supported by a series of protocols that enlisted licensed mental health professionals to make decisions about acute needs and ongoing referral. This included lines of communication within the program to mental health professionals as well as external supports from the research team who could arrange immediate assessment and hospitalization if necessary. According to the SCs in the focus groups, the construction of the decision aids and written communication protocols for decision-making somewhat allayed their liability concerns that had surfaced before in the focus groups.

The final deliverables were a modulized 6-hour educational presentation designed to meet the needs of non-mental health specialists (see Table 1) and a toolkit to support implementation of depression screening in mothers of children in EI services (see Table 2). All resources for use with parents were available in Spanish, with translation provided either by the publisher, as in the case of the screening tools, or by a professional translator employed by the research team. The toolkit was finalized after four iterations, incorporating feedback from the SCs and agency supervisors, and the education module included guidance on how to use the toolkit.

**Table 1.** Education Module Outline.

**WELCOME** ** Purpose** Review Early Intervention Mental Health Toolkit# Learn about maternal mental health with emphasis on depression and anxiety Skills to use with mothers with mental health symptoms *Recognizing Symptoms, Broaching the Topic, Screening for Symptoms, Discussing results, Supporting Mothers, and Monitoring Safety* **YOU ARE THE LIFELINE** Families are vulnerable You are a sensitive instrument in Early Intervention Your own self-care is crucial **THINGS YOU CAN DO** Recognize mental health symptoms *Differentiate between recognizing symptoms, screening, and diagnosing* *Symptoms of depression, variable presentations, and representative parenting interactions* * Symptoms of anxiety, variable presentations, and representative parenting interactions* Broach the topic *At enrollment: Normalizing* *Ongoing visits: Business as Usual* *Seize the moment: Share Something You Observe* Screen *Ask permission* *Use valid and reliable screening tools* Discuss results *Video example and role play* *Protocol and Decision tree* Plans *Emergency plan* *Safety Plan* *Referral Plan* Know your community resources Potential barriers 2-minute cases (role play) *Support Plan* Self-care Feelings cards Proactive Coping vs. Avoidant Coping 2-minute cases (role play) Monitoring Safety *Self-harm/Suicide* *Building a crisis management kit* *2-minute cases* (*role play*)

**Table 2.** Components of the Finalized Toolkit for Sites A and B.

Content	Source	Website
Scripts for introducing mental health issues and screening to parents	Researcher developed	Available through email contact
PHQ-9 with guidance on administering and scoring	Kroenke et al. (2001)	https://www.dartmouth-hitchcock.org/documents/questionnaire.pdf
GAD-7 with guidance on administering and scoring	Spitzer et al. (2006)	https://med.dartmouth-hitchcock.org/documents/GAD-7-anxiety-screen.pdf
Decision aid: Screening and referral	Researcher developed	Available through email contact
Decision aid: Crisis decision guide		
Parent resources available as handouts within toolkit		
Solving problems	Adapted from NIMH, VA, and SAMHSA websites	https://www.samhsa.gov/iecmhc/special-topics/maternal-depression and https://www.nimh.nih.gov/health/publications/depression/index.shtml
Managing stress		
Home safety		
When to call 911		
My plan (emergency/safety plan)		
Parents reactions to having a child with a developmental delay		
Staff and parent information handouts		
Customized annotated referral resource lists for each program service area	Researcher developed	Available through email contact

*Note.* All resources made available in English and Spanish. PHQ-9 = Patient Health Questionnaire-9; GAD-7= General Anxiety Disorder-7; NIMH = National Institute of Mental Health; VA = United States Department of Veterans Affairs; SAMHSA = Substance abuse and Mental Health Services Administration.

After the co-development of these deliverables, the SCs at site A received the training and began to execute the screening intervention. SCs collected data on the number of screenings they conducted and referrals made for the next year. Approximately 3 months after the conclusion of the trial at site A, the process was rolled out at site B by the senior investigator on the research team as well as the agency director. One SC declined participating because of personal reasons. The toolkit materials were updated with resources specific to site B’s catchment area. After the 6-hour training was conducted by the senior investigator, the research team members were less involved in the implementation at site B than at site A. This decision was made to replicate how we expected the toolkit and screening process to be implemented in other EI settings; a mental health interventionist would adapt the toolkit to the local agency and provide training and the agency would then take over the supervision of the SCs and depression screenings.

The reflection and evaluation phase began as soon as the first screenings started at site A. Internally, the agency director was available to the SCs as needed and collected their completed screenings on a weekly basis. The Director also formally followed up with the SCs as a group on at least a monthly basis with support, including logistical issues as well as clinical supervision. Clinical supervision appeared to be especially important, given the continued variation among SCs with conducting mental health screenings. The research team also conducted individual follow-ups with the SCs as requested to gain more insight into their perspectives on the implementation process. To encourage open reflection and evaluation of the integration of the screening process by the SCs, the specific content of these sessions was not shared with the regional director. Through these various avenues, SCs provided feedback on the major obstacles to their completion of screening, which were primarily their own discomfort and difficulty getting the parent alone to conduct a private screening. Multiple SCs reported that some parents expressed being glad that the SC brought up the issue of depressive symptoms and mental health to them. At site B, SCs indicated that the training was very helpful and had a positive response to the monthly supervision meetings where issues specific to each site (e.g., referral resources, differences in urban, and rural mental health referrals) were addressed with individual clients. After the conclusion of the monitored implementation at sites A and B, the agency director continued to be engaged on the research team for the remaining reflection and evaluation.

### Secondary Outcomes

In addition to evaluating the implementation according to the CFIR domains, our research team was interested in secondary outcomes of the implemented intervention (see Figure 1 and Table 3). At site A, there were 95 attempted screenings over the course of the year from a total caseload of approximately 175 families. The SCs reported that the screening took an average of 13 minutes. During the year at site A, over 10% of the mothers screened reported depressive symptoms above the threshold designated for referral. In all, 17 different referrals were made and 5 of those were accepted by the mother. The most commonly reported reason that mothers declined referrals was that they were already receiving mental health care. At site B, 12 screenings were attempted and completed out of an approximate caseload of 210 families. Again, over 10% (2 out of 12) of the mothers screened above the threshold for depressive symptoms, and one referral was made for psychiatric evaluation.

**Table 3.** Outcomes Following Implementation of Service Coordinator Screening for Depressive Symptoms.

Site	Screenings attempted	Screenings completed	Mothers scoring higher than 10 on PHQ-9	Average time spent screening	Referrals made	Referrals accepted
Site A	95	85	10	13 minutes	17 (for 14 adults)	5
Site B	12	12	2	Not reported	1	Not reported
Description of screening experiences by service coordinators						
1. Coordinators uncomfortable discussing mental health issues and screening parents; questioned whether parental mental health was appropriate for EI scope of work						
2. Parents were open to being screened, as many had been and were screened by their health providers						
3. Parents who declined to be screened stated that mental health issues were not concerning them at that time						
4. Service coordinators reported that a major obstacle was getting the parent alone to offer screening and providing a private place in which to complete the screening						
5. Parents who were screened once refused the second screening because they perceived their mood to be stable						

*Note.* PHQ9 = Patient Health Questionnaire-9; EI = Early Intervention.

## Discussion

Through our partnership with the SCs, we successfully completed the primary aims of the research project by developing the planned deliverables of (a) a comprehensive educational module and (b) a toolkit for implementing not only depression screenings but also for communicating with parents about mental health and facilitating referral and follow-up. To do so, the research team utilized focus groups to engage the SCs in a participatory process.

Yet, even with the development of these deliverables through a participatory process, the SCs at site A—the same ones who had developed the deliverables—found it challenging to conduct the screenings and reported mixed results. While prior studies found that approximately a third of mothers experienced significant depressive symptoms (Beeber et al., 2017; Feinberg et al., 2012), only about 10% of our samples screened as having a level of depressive symptoms warranting referral. Although it is possible that this reflects a true range of symptomatic mothers across studies, it also suggests to our research team that there may have been a number of mothers who were not screened who may have had significant depressive symptoms. The two main obstacles for completing screening reported by the SCs were (a) their own continued discomfort with discussing mental health issues with the parents, and whether these discussions were even within the scope of their practice and (b) providing a private place with the parent alone for screening. While we still believe that the preparatory collaborative work with our community partners in the specific setting was critical, and we suspect there would have been a much lower chance for success without their participation, a key takeaway from our experience is that the participatory process does not guarantee successful implementation of mental health screening.

As part of the CFIR model for intervention assessment, we assessed the characteristics of the individuals delivering the screenings. We found that the SCs overwhelmingly agreed that there was a need for these screenings but also that they did not feel comfortable being the ones conducting the screenings. This continued throughout the screening processes, despite a significant amount of expertise in psychiatric mental health by the research team which was translated into direct emotional health support for the SC team. The need for emotional health support for the SCs may indicate that future attempts at similar interventions would benefit from the integration of trauma-informed care by the research team for the SCs themselves. Anecdotally, it appeared that some SCs came to this work due to their own past experiences, leaving them more vulnerable to emotional taxation but also imbuing them with great passion and capacity to advocate on the part of the families they serve.

Through our assessment, we also identified that the individual SCs were the most significant difference between sites A and B rather than either the population or the rurality of the area served. At site A, the SCs had buy-in from the beginning of the project and had helped to design the training as well as the toolkit. At site B, the SCs supported the need for the screening but did not have that same level of vested interest as they were not involved as key stakeholders from the beginning. In addition, after the initial training, the research team was not directly engaged with the SCs at site B, which might have led to less performance bias in number of screenings completed.

In prior literature, Reynolds et al. (2012) reported greater success at integrating mental health screenings into EI services. Like our intervention, the researchers worked with SCs in their county; unlike our study, they also focused on engaging a behavioral health system and connecting the two systems through cross-system facilitators located within the EI offices. This weaving of behavioral health into the fabric of the intervention may have alleviated any trepidation that their SCs would have otherwise had, given that the role of the SCs was otherwise very similar between the two studies. However, given budget limitations, it would not have been possible for the agency that we partnered with to fund a similar cross-system facilitator to engage the behavioral health providers. The variability across states and municipalities of structure, funding, and motivation for integration of EI services with other resources is an inherent limitation to providing mental health screening services.

An additional barrier to using SCs for implementing mental health screenings was that the SCs only saw their clients once a month for progress assessments. This length of time before SCs would follow-up is a significant concern for the parents who are close to but do not meet the symptom-level criteria for referrals to mental health care.

Given these barriers, it would be reasonable to look elsewhere within the organization for others to step in, which we did as part of our assessment of the inner setting. Unfortunately, at least with this EI agency, no other individuals act as regular points of contact with families. Service providers, who may have more advanced training and thus feel more competent with screening the mothers, were all contracted through external agencies and thus no coordinated efforts to introduce screening were possible.

Still, despite the challenges to implementing the screening itself, the education and training likely improved the SCs’ awareness of mental health issues and their ability to pick up symptoms of depression. If they did decide to screen, the toolkit provided the comfort of a protocol, including a list of possible resources that they could choose from as referrals. This alone may make it easier for the SCs to screen as they do not have to locate a tool, decide what score is worrisome or requires referral or immediate action, and do not have to spend time searching for available resources for referral.

## Conclusion

This study provides an exemplar for implementing screening for mental health issues in EI. By using the CFIR to guide us, we were careful to include the critical steps of listening and collaborating with those who were asked to change their practice in a meaningful and in-depth way. Given the variation in the way EI services are provided across localities, states, and the nation, using the CFIR to guide assessment, evaluation, and ultimately, development of a contextually tailored toolkit and protocol is highly recommended for future projects. The partnership and collaboration between the diverse and highly experienced research team and the agency staff was critical. The core components of the intervention, screening, identification referral, resource provision and follow-up—which must remain consistent—were supported by adapting the education and toolkit to the local setting. It is especially important that each agency be prepared to develop their own resources and referrals for the families they serve.
